# RNA N6-methyladenosine (m6A) regulates cell cycle progression in diffuse midline glioma (DMG) and confers sensitivity to FTO inhibition

**DOI:** 10.1038/s41419-026-08647-8

**Published:** 2026-03-26

**Authors:** Samuel E. Ross, Holly Holliday, Eyden Wang, Mahdi Zeraati, Maria Tsoli, David S. Ziegler, Marcel E. Dinger

**Affiliations:** 1 School of Life and Environmental Sciences, University of Sydney, Sydney, NSW Australia; 2 Children’s Cancer Institute at Minderoo Children’s Comprehensive Cancer Centre, Sydney, NSW Australia; 3 School of Clinical Medicine, UNSW Sydney, Sydney, NSW Australia; 4 Kid’s Cancer Centre, Sydney Children’s Hospital, Randwick, NSW Australia

**Keywords:** Paediatric cancer, RNA modification, Epigenetics, Transcriptomics

## Abstract

Diffuse midline gliomas (DMG) are deadly pediatric brain cancers with limited treatment options. These tumors likely arise from oligodendrocyte precursor cells (OPC) that acquire a driver histone mutation, leading to an aberrant epigenome. RNA N6-methyladenosine (m6A) is a vital epi-transcriptomic modification that regulates RNA processes and plays a significant role in OPC development through its regulation of transcripts involved in histone modification processes. Despite this pivotal role in OPC biology, the epi-transcriptome has not yet been investigated in DMG, and its interrogation may uncover new therapeutic options and understanding of this disease. Therefore, for the first time, we generated base-resolution m6A landscapes for patient-derived DMG cultures and found that DMG exhibits elevated m6A levels compared to non-neoplastic patient cells, with particularly strong enrichment on transcripts involved in cell motility and migration. In contrast, the minority of transcripts that have lower levels of m6A in DMG were associated with cell cycle regulation, especially components of chromosome segregation machinery. We also demonstrate that DMG is sensitive to inhibition of the m6A demethylase FTO, with FB23-2 treatment resulting in decreased proliferation, reduced survival, and pronounced S-phase arrest/stress, accompanied by robust induction of CDKN1A, GADD45B, and TFRC. Furthermore, FTO inhibition led to significant downregulation of key cell cycle regulators at both the transcriptomic and proteomic levels. Collectively, these findings highlight RNA methylation as a critical regulator of DMG tumorigenicity and identify FTO as a promising therapeutic target for this currently incurable disease.

## Introduction

Diffuse midline glioma H3 K27-altered (DMG), is a highly aggressive brain tumor that is believed to derive from oligodendrocyte precursor cells (OPC) [1–3]. DMG is predominantly diagnosed in children under the age of 10 and is characterized by either the recurrent lysine-to-methionine driver mutations in Histone H3.1 and H3.3 genes (H3K27M), or overexpression of EZHIP [4–10]. These drivers lead to an aberrant epigenetic landscape with loss of Histone H3K27 trimethylation (H3K27me3), increased H3K27 acetylation (H3K27ac) and DNA hypomethylation [10–16]. While substantial advances have been made in elucidating the epigenetic dysregulation in DMG, the epi-transcriptome – a regulatory layer involving chemical modifications to RNA – remains largely unexplored.

RNA N6-methyladenosine (m6A) is the most abundant epi-transcriptomic modification and is found on both mRNA and non-coding RNA [17–19], and plays roles in regulating RNA stability, transport, splicing, translation, and protein-RNA interactions [20–25]. On mRNA, m6A is enriched at DRACH motifs (D = A, G or U; H = A, C or U) [26, 27], near stop-codons and 3’ untranslated regions (UTR), and is deposited by METTL3 [28–32]. Cross-talk between m6A deposition and H3K27 and H3K36 methylation levels have also been reported [33–36], which may be relevant to DMG where both H3K36me2 and H3K27me3 are aberrantly distributed [15, 16, 37, 38].

M6A can also be actively removed from RNA by two demethylases: Fat Mass and Obesity Associated (FTO) and AlkB homolog 5 (ALKBH5) [39–41]. Additionally, m6A can be recognized by reader proteins such as YTH N6-Methyladenosine RNA Binding Proteins1/2/3 (YTHDF1/2/3), YTH Domain Containing 1/2 (YTHDC1/2), and Insulin-like Growth Factor 2 mRNA-Binding Proteins 1/2/3/ (IGF2BP1/2/3) [20, 42–47]. Interestingly, YTHDF2 is overexpressed in DMG [48] and is the only reader with a well-defined role in the m6A-dependent degradation of transcripts [24, 47]

Recent developments of small molecule inhibitors for METTL3 and FTO have revealed that targeting m6A dynamics can have therapeutic benefits in cancers by regulating proliferation, self-renewal, and survival [49–54]. Moreover, m6A is essential for neural stem cell identity [55] and oligodendrocyte maturation [56], partly through its regulation of transcripts encoding histone-modifying enzymes [55, 56]. Considering the biological roles of m6A, the origin of DMGs from OPCs, and the presence of a disruptive oncogenic histone mutation, we aimed to explore the m6A landscape in DMG.

For the first time, we characterize the m6A landscape of patient-derived DMG cultures at base resolution and reveal that they exhibit elevated m6A levels compared to patient-derived normal cells. The most pronounced increases occur on transcripts associated with cell migration and epithelial-to-mesenchymal transition, whereas the minority of transcripts with decreased m6A levels in DMG are enriched for functions related to chromosome segregation and cell cycle regulation. We further reveal that DMG cultures are sensitive to FTO inhibition, and not METTL3 inhibition, with FB23-2 treatment resulting in decreased survival, cell cycle dysregulation, and profound transcript and protein level changes. Ultimately, this study provides the first detailed investigation of the m6A landscape in DMG, reveals an association between m6A and DMG tumorigenicity, and highlights FTO inhibition as a potential therapeutic strategy for this currently incurable disease.

## Results mapping the RNA N6-methyladenosine (m6A) landscape of DMG cells

**Fig. 1:**
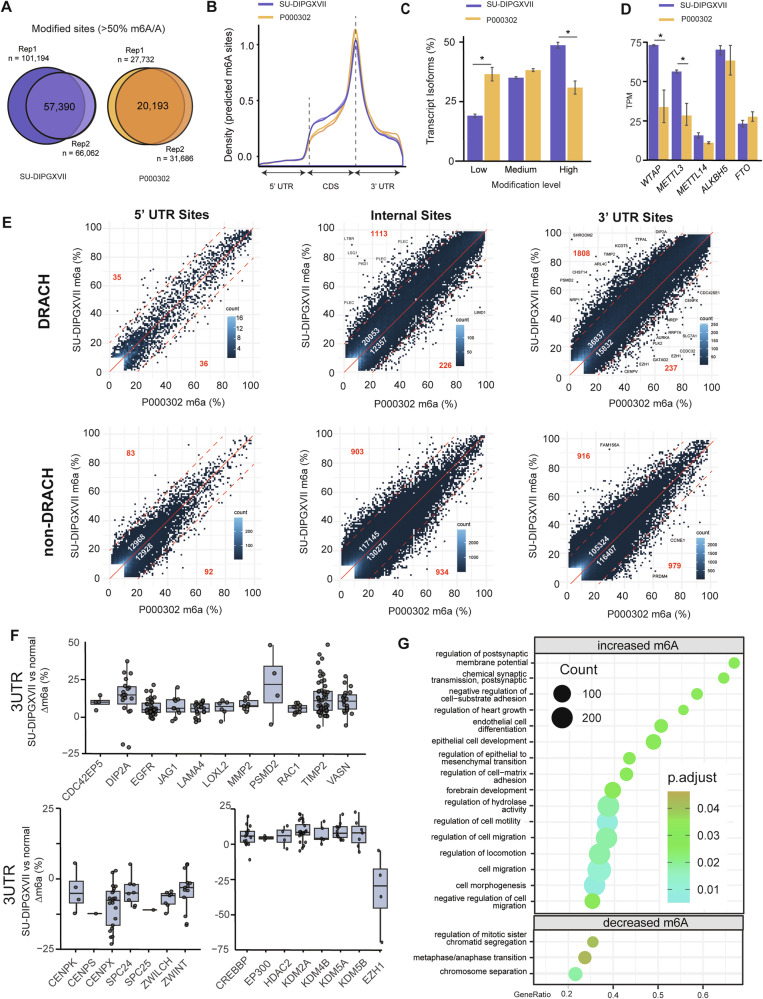
RNA N6-Methyladenosine (m6A) landscape of DMG cells vs non-neoplastic, patient-derived brain cells. **A** Overlap of m6A sites between biological replicates of SU-DIPGXVIII and a patient-derived non-neoplastic brain cell culture (P000302) (>50% m6A/A; >10 reads). **B** Density plot showing the distribution of m6A sites (>50% m6A/A; >10 reads) across transcripts. **C** Percentage of transcript isoforms with low (<30% of reads), medium (30–70%), or high (>70%) levels of m6A modifications. **D** Expression levels (TPM) of genes involved in m6A deposition and removal. Error bars represent standard error. **E** Scatterplots showing m6A levels at transcript sites in SU-DIPGXVII compared to P000302 across the 5′UTR, CDS (internal), and 3′UTR. Sites are separated into DRACH (top) and non-DRACH (bottom). White numbers: number sites with >10% difference (above/below red solid line). Red numbers: sites with >%20 difference (above/below red dotted line). Sites are filtered for >10 coverage and >10% average m6A in one condition. **F** Difference in m6A levels (SU-DIPGXVII m6A% – P000302 m6A%) at DRACH motifs in the 3UTR (+100 bp) of transcript involved in cell motility and migration (top), chromosome separation (bottom left) and histone modifications (bottom right). Sites are filtered for >10 coverage and >10% average m6A in one condition. **G** Gene set enrichment analysis of genes ranked by the average m6A difference across all modified sites (>10× coverage; >10% m6A in at least one condition) between SU-DIPGXVII and P000302.

To investigate m6A levels in DMG, we generated direct RNA sequencing libraries (RNA004 chemistry) from a patient-derived DMG (SU-DIPGXVII) and a non-neoplastic, patient-derived brain cell culture (P000302) [57]. We observed a high degree of overlap in m6A-modified sites (m6A/A > 50%) between biological replicates (Fig. 1A). Despite the distinct epigenomic landscape of DMG, visualization of m6A site distribution revealed a canonical pattern in both culture types, with m6A-modified sites enriched near the stop codon and within the 3′ UTR of transcripts (Fig. 1B).

We next quantified m6A modification levels across transcript isoforms, and defined isoforms as having either low levels of m6A (<30% of reads modified), medium levels (30-70% reads modified) or high levels (>70% reads modified). SU-DIPGXVII displayed a significantly higher proportion of highly modified isoforms (47% vs 30% of isoforms) and a significantly lower proportion of lowly modified isoforms (18% vs 35%)(Fig. 1C). Notably, this elevation in m6A levels coincides with increased expression of m6A writer complex components (*METTL3*, *WTAP*, and *METTL14*) in the DMG culture (Fig. 1D). Elevated m6A levels have also been reported in neural stem cell populations, where they support maintenance of cellular identity and self-renewal [55, 56], a function which may be conserved in DMG cells here.

To further investigate these elevated m6A levels in DMG versus normal cells, we analyzed modification levels at all individual sites with >10 reads per sample (Supplementary Table 1). We found that m6A levels were comparable between the two lines at non-DRACH motifs and within 5′ UTRs. In contrast, DRACH motifs within the Coding Sequence (CDS) and 3′ UTRs showed a pronounced increase in m6A levels in DMG with 2922 sites exhibiting >20% higher m6A levels compared to only 463 sites in the non-neoplastic cells (Fig. 1E).

Interestingly, among the most differentially hypermethylated sites in DMG were numerous genes involved in cell motility, invasion, and epithelial-to-mesenchymal transition (EMT), including *SHROOM2, PSMD2, TTPAL, TIMP2, NRP1*, and *KCDT5* (Fig. 1E). In contrast, hypomethylated sites were enriched for genes regulating cell cycle progression (*CENPX, CENPV, AURKA, PLK2*) and for *EZH1*, a H3K27me3 methyltransferase inhibited in DMG by the H3K27M mutation. Additionally, multiple genes displayed significant changes in m6A levels across many sites in their 3′ UTR, including genes involved in motility/migration and cell cycle regulation (Fig. 1F).

Given the hypomethylation observed within the EZH1 3′ UTR, and previous reports linking m6A and histone modifiers to oligodendrocyte precursor cell (OPC) differentiation, we then also examined m6A levels in key regulators relevant to DMG, including *CREBBP* and *EP300* (H3K27ac), *EZH1* (H3K27 methylation), and several *KDM* enzyme transcripts (H3K36, H3K4, and H3K9 demethylation). Strikingly, the 3′ UTRs of all these histone regulators were hypermethylated in DMG, with the exception of *EZH1*, whose function is suppressed in DMG (Fig. 1F).

Finally, gene ontology enrichment analysis was performed after ranking genes by the average difference of all modified sites (>10% modified; >10× coverage) and revealed strong enrichment for terms related to migration, motility, EMT, forebrain development, and chromosome segregation, consistent with the observations made from individual site changes in 3′ UTRs (Fig. 1G). Together, these findings suggest that m6A plays important roles in regulating DMG biology and that targeting m6A may represent a promising therapeutic strategy.

## Increased METTL3 is associated with the stem-like state of DMG

**Fig. 2:**
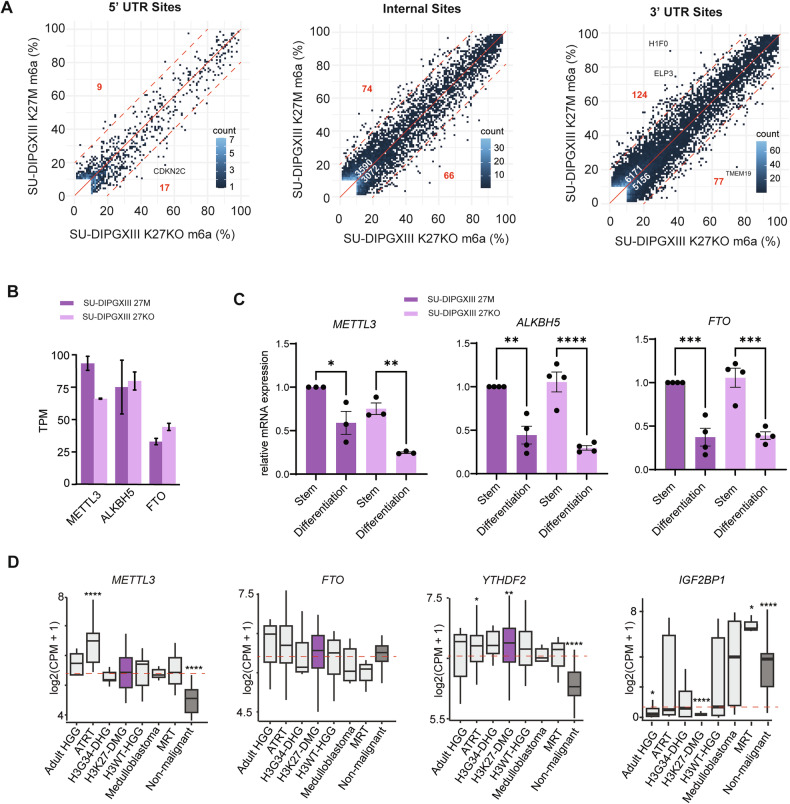
Landscape of m6A and its regulators in DIPGXIII and its isogenic lines. **A** Scatterplots of DRACH m6A levels in SU-DIPGXIII (K27M) versus its isogenic K27M-knockout line across the 5′UTR, CDS (internal), and 3′UTR. White numbers: number sites with >10% difference (above/below red solid line). Red numbers: sites with >%20 difference (above/below red dotted line). Sites are filtered for >10 coverage and >10% average m6A in one condition. **B** Expression levels (TPM) of *METTL3*, *ALKBH5* and *FTO* in SU-DIPGXIII and its isogenic lines. Error bars represent standard error. **C** Relative expression levels of *METTL3, ALKBH5*, and *FTO* in SU-DIPGXII and its isogenic line after 3 days of culturing in stem vs differentiation media. Error bars represent standard error. *p < 0.05, **p < 0.01, ***p < 0.001, ****p < 0.001. **D** Average transcript levels (log2(CPM + 1) of m6A readers in different cancers and non-malignant cells [48]. Adult high-grade gliomas (Adult HGG; *n* = 16), typical teratoid rhabdoid tumors (ATRT; *n* = 19), H3G34-mutant diffuse hemispheric gliomas (H3G34-DHG; *n* = 6), H3K27-altered diffuse midline gliomas (H3K27-DMG; *n* = 51), H3WT - high grade gliomas (H3WT-HGG; *n* = 19), Medulloblastoma (*n* = 7), malignant rhabdoid tumors (*n* = 7), non-malignant (*n* = 39). The boxplots show the median, first and third quartiles with the whiskers extended to the last point within 1.5X of the interquartile range. Red dotted line indicates cohort median. *P*-value for significant difference from median shown: *<0.05, **<0.01, ***<0.001, ****<0.0001.

To determine whether the H3K27M mutation influences the m6A landscape we identified, we generated direct-RNA sequencing libraries (RNA002 chemistry) from the patient-derived DMG culture SU-DIPGXIII and its isogenic SU-DIPGXIII K27KO line, which lacks the H3K27M mutation [13, 58]. We found no substantial differences between their m6A landscapes, other than a minor increase in m6A levels at 3′ UTRs in SU-DIPGXIII (124 sites with >20% increase vs. 77 sites with >20% decrease; Fig. 2A). However, we did detect a ~50% increase in *METTL3* expression in SU-DIPGXIII compared to K27KO (Fig. 2B).

To assess whether the elevated m6A profile may instead reflect the OPC-like state of DMG, we examined expression of m6A writers and erasers under differentiation conditions [3]. Under differentiation conditions, both lines showed significant reductions in *METTL3*, *FTO*, and *ALKBH5* expression (Fig. 2C). These results suggest that the elevated m6A landscape in DMG is driven primarily by the stem-like state.

We next compared expression of m6A regulators in DMG to other pediatric tumors, adult brain tumors, and non-malignant tissues [48]. Among m6A writers and erasers, *METTL3* showed the strongest elevation in cancer samples relative to non-malignant cells, consistent with its association with proliferative states (Fig. 2D). In contrast, the m6A reader IGF2BP1, known to play oncogenic roles in other cancers [59], displayed the lowest expression in DMG compared to all other tumor types, however YTHDF2, a key mediator of m6A-dependent mRNA degradation [20, 47], showed the highest expression. Together, these features further support important and unique roles of m6A regulation in DMG and reinforce it as a potential therapeutic target.

## FTO inhibition causes cell cycle dysregulation leading to decreased proliferation and survival in DMG

**Fig. 3:**
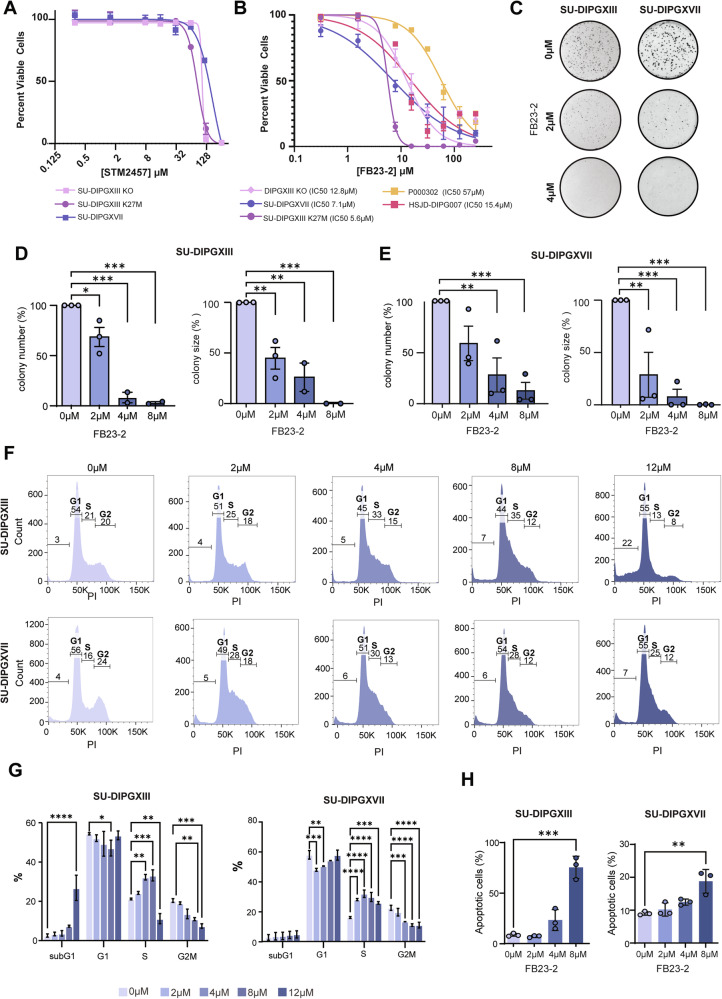
Effects of FB23-2 treatment on DMG survival, proliferation, and cell cycle progression. **A** Viability of cell lines following 24 h of STM2457 treatment (0.3 µM, 1.6 µM, 7.8 µM,15.6 µM, 31.2 µM, 62.5 µM, 125 µM, 250 µM). Data is represented as the mean of 3 biological replicates with standard error shown. Patient-derived DMG lines: SU-DIPGXVII and SU-DIPGXIII; Isogenic K27M KO: SU-DIPGXIII K27M KO. **B** Viability of cell lines following 24 h of FB23-2 treatment (0.3 µM, 1.6 µM, 7.8 µM,15.6 µM, 31.2 µM, 62.5 µM, 125 µM, 250 µM). Data is represented as the mean of 3 biological replicates with the standard error shown. Patient-derived DMG cultures: SU-DIPGXVII, HSJD-DIPG007, SU-DIPGXIII; Isogenic K27M KO: SU-DIPGXIII K27M KO; Patient-derived, non-neoplastic P000302. **C** Represented images of colony-forming assays performed on patient-derived DMG lines (SU-DIPGXVII and SU-DIPGXIII) following 72 h of FB23-2 treatment (0–4 µM). **D** Colony number (left) and size (right) of SU-DIPGXIII and **E** SU-DIPGXVII, following 2 days of FB23-2 treatment (0–8 µM). Data is represented as the mean of 3 biological replicates with the standard error shown. *p < 0.05, **p < 0.01, ***p < 0.001. **F** Flow cytometry analysis for cell cycle distribution of SU-DIPGXIII (Top) and SU-DIPGXVII (bottom) following 24 h treatment with FB23-2 (0–12 µM). **G** Quantification of cell cycle states of DMG cells following 24 h treatment with FB23-2 (0–12 µM) in SU-DIPGXIII and SU-DIPGXVII. Data is represented as the mean of 3 biological replicates with the standard error shown. *p < 0.05, **p < 0.01, ***p < 0.001, ****p < 0.001. **H** Flow cytometry analysis for apoptosis of DMG cells following 24 h treatment with FB23-2 (0–8 µM) in SU-DIPGXIII and SU-DIPGXVII. Data is represented as the mean of 3 biological replicates with the standard error shown. *p < 0.05, **p < 0.01, ***p < 0.001.

Given these results, we next aimed to explore the impact of altering m6A dynamics on DMG proliferation, survival, and cell cycle progression. Cell viability assays were performed on three DMG cultures (SU-DIPGXIII, SU-DIPGXVII, HSJD-DIPG007 [60]), the non-neoplastic patient-derived line (P000302), and an isogenic H3K27M KO DMG line (SU-DIPGXIII KO), lacking H3K27M [13, 58]. Cell viability assays were performed following treatment with STM2457, a METTL3 inhibitor [54], or FB23-2, an FTO inhibitor [51]. Surprisingly, STM2457 treatment had minimal effect on DMG cell survival, with negligible effect on cell viability detected up to 125 µM (Fig. 3A). For context, STM2457 has previously been shown to inhibit AML proliferation at doses of ~1 µM [54]. However, FB23-2 treatment had pronounced effects on DMG cell survival with IC_50_ values of 5.6, 7.1, and 15.4 µM for the DMG cultures SU-DIPGXIII, SU-DIPXVII, and HSJD-DIPG007, respectively (Fig. 3B). The IC_50_ value for the isogenic “WT” DMG culture was 12.8 µM which was lower than its corresponding mutant SU-DIPGXIII but comparable to the HSJD-DIPG007 (Fig. 3B). These IC_50_ value ranges observed for DMG are similar to the values observed for AML cell lines (2–5 µM), for which FB23-2 was originally developed and synthesized [51]. Moreover, the IC_50_ value for the non-neoplastic line (P000302) was substantially higher at 57 µM (Fig. 3B). These results indicate that DMG cells are sensitive to FB23-2 treatment and FTO inhibition with a potentially wide therapeutic window. We then further validated the response of the most sensitive line to FTO inhibition using siRNA-mediated knockdown, which resulted in an ~40% decrease in cell confluency with a corresponding ~66% reduction in FTO transcript levels (Supplementary Fig. 1A–C).

To further elucidate the effects of FTO inhibition on DMG cells, we next performed colony-forming assays after FB23-2 treatment on the two most sensitive DMG cultures. FB23-2 treatment significantly reduced colony numbers and colony sizes for SU-DIPGXIII and SU-DIPGXVII, at concentrations above 2 uM and 4 µM, respectively (Fig. 3C–E). Lastly, we investigated the effects of FB23-2 treatment on cell cycle progression and apoptosis. We observed an unusual and striking increase of cells in the S-phase and a decrease of cells in G2/M, in a dose-dependent manner (Fig. 3F, G). For example, at 4 µM FB23-2 treatment, we observe an almost 50% increase in S-phase cells for SU-DIPGXIII and an almost 100% increase for SU-DIPGXVII (Fig. 3F, G). However, we observed that at 12 µM treatment in SU-DIPGXIII, there is then a potent reduction of cells in the S-Phase and a concurrent increase in subG1 cells (Fig. 3F, G). This is consistent with observations in AML [51], including the S-phase arrest following treatment with Brequinar [53]. Finally, analysis of apoptosis by flow cytometry revealed that these cells do undergo a significant increase in apoptosis at these higher drug doses (Fig. 3H; Supplementary Fig. 1D). Together, these results indicate that FB23-2 treatment results in a prolonged or stalled S-phase followed by apoptosis.

Overall, we find DMG cells are sensitive to FTO inhibition with FB23-2 treatment resulting in cell cycle dysregulation, decreased proliferation, induction of apoptosis and decreased survival. Based on these results and the m6A landscape, we hypothesize that DMG cells are sensitive to FTO inhibition as they rely on FTO to tightly regulate transcript stability during their rapid cell cycle.

## FTO inhibition causes gene expression changes in cell cycle and apoptotic pathways in DMG

**Fig. 4:**
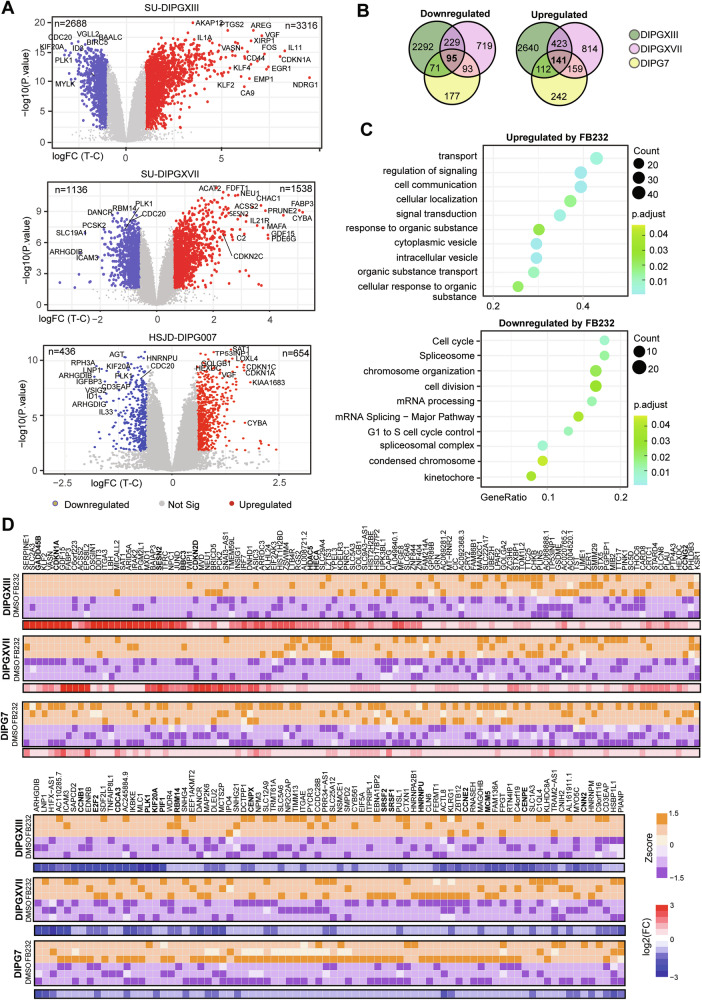
Gene expression changes following FB23-2 treatment of DMG cells. **A** Volcano plots depicting gene expression changes in SU-DIPGXIII (top), SU-DIPGXVII (middle) and HSJD-DIPG007 (bottom) following 24 h treatment with ~IC50 doses of FB23-2 (8 µM for SU-DIPGXIII and SU-DIPGXVII, 10 µM for HSJD-DIPG007). Blue dots represent significantly downregulated genes (FDR < 0.05, FC < −1.5) and red dots for significantly upregulated genes (FDR < 0.05, FC > 1.5). **B** Overlap between genes differentially downregulated (left) and upregulated (right) in SU-DIPGXIII, SU-DIPGXVII and HSJD-DIPG007 after FB23-2 treatment. **C** Gene Ontology enrichment analysis of genes commonly upregulated and downregulated in DMG cell lines after FB23-2 treatment. **D** Heatmap of log2FC and Zscore values for genes that are significantly upregulated and downregulated (FDR < 0.05, log2FC > 0.58) in all three DMG cultures following FB23-2 treatment.

To explore the effects of FTO inhibition on gene expression and identify putative transcripts with m6A-dependent regulation, we next performed RNA-seq on the three DMG cultures (HSJD-DIPG007, SU-DIPGXIII, SU-DIPXVII) after 24-hour treatment with ~IC50 doses of FB23-2. All three lines displayed significant levels of gene expression changes following FB23-2 treatment (FDR > 0.05; FC > 1.5) (Fig. 4A). Interestingly, some of the most significantly changed genes in all lines were cell cycle regulators, such as *PLK1, KIF20A, CDC20, CDKN1A*, and *CDKN2C* (Fig. 4A; Supplementary Table 2). Notably, SU-DIPGXIII was the most sensitive line to FB23-2 treatment and had a staggering 270-fold increase in *CDKN1A* expression.

To find common transcripts dysregulated after FB23-2 treatment, we next looked at differentially expressed genes that overlapped in all three cell lines and found 96 commonly downregulated transcripts and 141 commonly upregulated transcripts (Fig. 4B; Supplementary Table 2). Gene ontology enrichment analysis of these genes revealed terms associated with cell cycle regulation, G1 to S cell cycle control, and kinetochore for downregulated genes following treatment (Fig. 4C). Importantly, these pathways are highly similar to those identified as having reduced m6A in DMG compared to patient-derived, non-neoplastic brain cells (Fig. 1G) and are consistent with the cell cycle dysregulation observed in the cellular assays (Fig. 3F, G). These genes included vital cell cycle machinery and regulators such as *CCNB1, CCNE2, E2F2, PLK1, KIF20A, CDCA3, RBM14, CENPX, CENPE, HNRNPU*, and *MCM5* (Fig. 4D). Meanwhile, some of the most significantly upregulated genes were associated with DNA stress/damage such as *CDKN1A, CDKN2D, GADD45B, TFRC, SESN2, BBC3*, and *HECA* (Fig. 4D). Based on these results together with the cellular assays, we hypothesize that FB23-2 treatment causes reduced levels of positive cell cycle regulators /machinery leading to stress and the induction of transcripts such as *CDKN1A*, *GADD45B*, and *TFRC*. This hypothesis is supported by previous reports that FTO can regulate cell cycle progression [61] and that *CDKN1A* is known to be upregulated after S-phase stress to promote cell cycle arrest [62].

We then performed RNA-seq on the isogenic SU-DIPGXIII K27M knockout (27KO) line treated with FB23-2 to explore whether the observed gene expression changes were associated with the H3K27M mutation. We observed a strong overlap in differentially expressed genes between the parental and isogenic lines, including enrichment of chromosome segregation as a top term among downregulated genes (Supplementary Fig. 2A, B). This included common and significant downregulation in many key kinetochore and chromosomal machinery components (Supplementary Fig. 2C).

However, the parental SU-DIPGXIII line did display substantially more differentially expressed transcripts in response to FB23-2 compared to the isogenic 27KO (Supplementary Fig. 2A), and had upregulated genes enriched for processes related to motility, migration, and angiogenesis (Supplementary Fig. 2B). Notably, these same pathways showed the greatest increase in m6A modification in DMG versus non-neoplastic cells, suggesting that FTO inhibition could influence migration and invasion.

Finally, we also wished to explore how FTO inhibition effected the expression of lineage factors and histone modifiers associated with OPC maturation, as this too may contribute to the changes in cell cycle dynamics and growth. These analyses identified key OPC/neural stem-cell factors such as *NES, ID1-3, OLIG1, NKX2.2, HES5, SOX3, DLX1*, and *HOXD3*, to be downregulated after FTO inhibition in at least 1 cell line, with similar trends in all (Supplementary Fig. 2D). This, along with the decreased levels of FTO during DMG cells cultured in differentiation conditions (Fig. 2C), suggests FTO levels plays a role in maintaining stemness. Consistently, we observed a modest decrease in stem cell factors NES and SOX2 after treatment (Supplementary Fig. 2E; Supplementary Material). However, morphological changes associated with differentiation could not be assessed due to the rapid morphological deterioration of cells after treatment.

In summary, FTO inhibition induces widespread transcriptional changes in DMG cultures, particularly affecting cell cycle and stress-response pathways. The reduced m6A levels on key cell cycle regulators further suggest that FTO may directly influence cell cycle progression by modulating m6A and the regulation of these transcripts.

## Effects of FB23-2 treatment on the m6A landscape of DMG

Given our earlier observation that the m6A reader YTHDF2 is overexpressed in DMG (Fig. 2D) and that many cell cycle regulators were downregulated by FB23-2 treatment, we next explored whether this could be due to YTHDF2-dependent degradation. Therefore, we investigated which commonly dysregulated genes had both m6A modifications and previously characterized YTHDF2 binding sites from Glioblastoma stem cells (GSC) [63] or Hela cells [20]. Interestingly, we find ~14% of commonly downregulated genes have m6A modification and YTHDF2 binding sites determined from GSC experiments compared to only ~2% of commonly upregulated genes. Strikingly, the m6A-modified and YTHDF2-bound *PLK1*, *KIF20A*, and *MLC1* transcripts are also the three most downregulated genes in SU-DIPGXIII (Supplementary Table 2). Moreover, we find the two most sensitive FB23-2 cultures (SU-DIPGXIII and SU-DIPXVII) to have high and comparable expression levels of *YTHDF2*, while the least sensitive (HSJD-DIPG007) had ~30% lower levels (Supplementary Fig. 2F). We also observed that SU-DIPGXIII displayed the highest expression of the m6A regulator*s FTO, METTL3, and WTAP* (Supplementary Fig. 2F). Together, these data suggest that the sensitivity of DMG cells to FTO inhibition may be influenced by the expression levels of key m6A regulatory components, including YTHDF2.

**Fig. 5:**
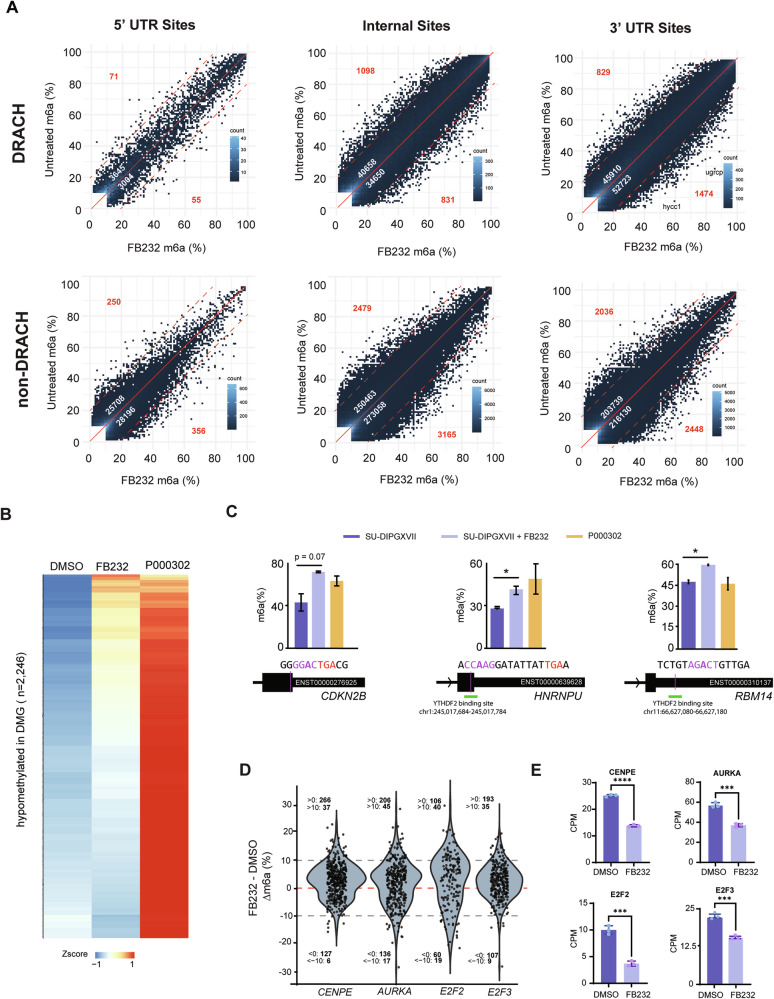
Effects of FB23-2 treatment on the m6A landscape in SU-DIPGXVII. **A** Scatterplots showing m6A levels at transcript sites in FB23-2 treated vs untreated SU-DIPGXVII cells after 24 h. White numbers: number of sites with >10% difference (above/below red solid line). Red numbers: sites with >%20 difference (above/below red dotted line). Sites are filtered for >10 coverage and >10% average m6A in one condition. **B** Heatmap showing m6A levels at sites hypomethylated in SU-DIPGXVII compared to non-neoplastic patient cells in untreated SU-DIPGXVII, FB23-2 treated SU-DIPGXVII, and P000302. Values are represented as Zscores. **C** m6A levels at a DRACH site in *CDKN2B*, a non-DRACH site overlapping a YTHDF2 binding site [63] in *HNRNPU*, and a DRACH site overlapping a YTHDF2 binding site [20] in *RBM14*. Error bars represent standard error. Genomic coordinates given for hg38 assembly. **D** Violin plots showing m6A level difference (m6A% treated – m6a% untreated) across all sites in *CENPE, AURKA, E2F2*, and *E2F3*. Sites are filtered for >10 coverage and minimum 10% average m6A in one condition. **E** Expression levels (CPM) of *CENPE, AURKA, E2F2*, and *E2F3* in FB23-2 treated vs untreated SU-DIPGXVII after 24 h. Data is represented as the mean of 3 biological replicates with the standard error shown. *p < 0.05, **p < 0.01, ***p < 0.001, ****p < 0.0001.

To identify direct transcriptomic effects of FTO inhibition in DMG, we performed direct RNA sequencing on SU-DIPG-XVII cells (an intermediately sensitive line) following FB23-2 treatment [Supplementary Table 3]. While most sites showed minimal differences upon treatment, we detected that at DRACH motifs within 3′ UTRs, the treated samples displayed a greater proportion of relatively hypermethylated sites, with 1474 sites exhibiting a >20% increase in m6A compared to untreated cells (Fig. 5A). This represents ~50% more hypermethylated sites than observed in the untreated samples (829). Interestingly, we also observed a modest increase in non-DRACH m6A sites, with ~25% more sites showing relative hypermethylation in treated versus untreated cells (5969 vs. 4765) (Fig. 5A). This modest shift is consistent with previous reports showing that FTO inhibition induces only minor global increases in m6A abundance (~10–20%) [51], which may reflect both the restricted substrate specificity of FTO and its differential activity across cell-cycle phases, only some of which are captured within the pooled RNA.

We next investigated m6A levels at all sites we had found to be hypomethylated in DMG vs normal cells to determine if this could be due to differential FTO activity. Indeed, we found that ~25% of these sites showed full or partial restoration of m6A levels following FTO inhibition (*n* = 571, >10% m6A restored) (Fig. 5B). Notably, these sites included a DRACH motif at the *CDKN2B* stop codon and a non-DRACH sites within the 3′UTR region of *HNRNPU* which overlaps with a high-confidence YTHDF2 binding site [20] (Fig. 5C). Additionally, we also noted other sites within YTHDF2 binding sites which showed an exclusive increase in m6A levels in the treated samples, such as for *RBM14* (Fig. 5C). Notably, all these transcripts are among the most significantly dysregulated following FB23-2 treatment (Supplementary Table 2).

To detect subtle treatment-induced m6A changes that may reflect cell cycle-dependent FTO activity, we calculated a net m6A change per gene by comparing the number of sites showing any increase versus decrease in methylation. Using this approach, we found that the top 100 genes with the highest net-positive m6A gain (filtered for RNA-seq FDR < 0.01) were markedly enriched for downregulated genes (*n* = 32), with only a small number being upregulated (*n* = 3) after FB23-2 treatment in SU-DIPGXVII. This included key regulators of cell cycle and chromosome separation *CENPE, AURKA, E2F2, E2F3* (Fig. 5D, E).

Overall, these results again indicate that FTO may directly regulate cell cycle progression via m6A levels in DMG, particularly by modulating transcript stability during different stages. Notably, FTO KO has also been shown to cause chromosome instability [64], and recent work has linked m6A levels to centrosome integrity [65].

## FTO inhibition induces protein-level changes in cell cycle and apoptosis pathways

**Fig. 6:**
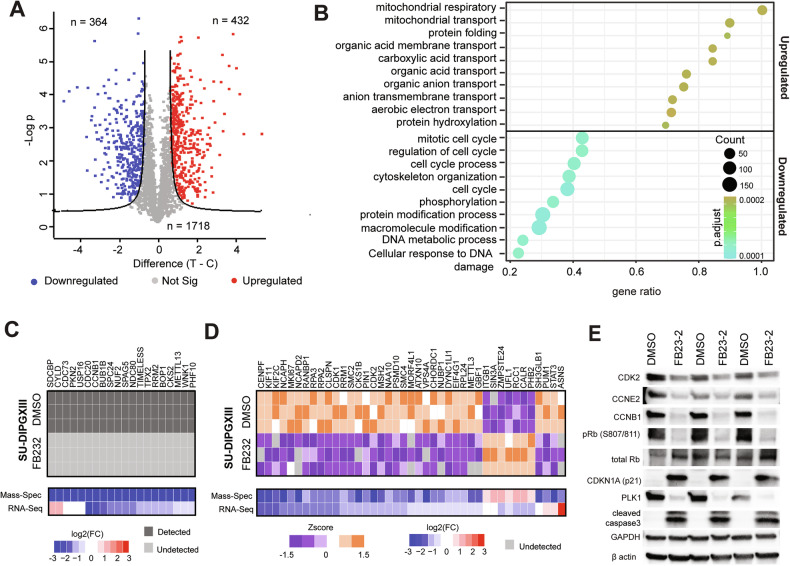
Proteomic analysis of FB23-2-treated DMG cells. **A** Volcano plots depicting protein level changes in SU-DIPGXIII cells following 24 h treatment with 8 µM FB23-2. Blue dots represent significantly downregulated proteins (FDR < 0.05, FC < −2) and red dots represent significantly upregulated proteins (FDR < 0.05, FC > 2). **B** Gene Ontology enrichment analysis of proteins with differential abundances after FB23-2 treatment in SU-DIPGXIII. **C** Heatmaps of proteins involved in cell cycle regulation (GO:0051726), which were detected in all three DMSO-treated SU-DIPGXIII replicates but not in any FB23-2-treated replicates. log2FC values of the corresponding transcript from RNA-seq experiments are also shown. log2FC values for mass-spectrometry experiments taking as <−3 if proteins were undetectable in FB23-2 treated samples. **D** Heatmap of log2FC and Zscore values for proteins involved in cell cycle regulation (GO:0051726), which had differential levels after 24 h treatment with 8 µM FB23-2 in SU-DIPGXIII. Log2FC values for the corresponding transcript from RNA-seq experiments are also shown. **E** Western blots analysis for cell cycle and apoptosis regulators following 24 h treatment of 8 µM FB23-2 in SU-DIPGXIII (*n* = 3). pRb phosphorylated Rb (retinoblastoma protein), S807/811 serine 807/811.

As m6A modifications also affect transcript export and translation, we investigated protein-level changes following FB23-2 treatment. To that end, we performed mass spectrometry on SU-DIPGXIII cells after 24-hour treatment with 8 µM FB23-2 (Supplementary Fig. 3A). Out of the ~2500 proteins that were confidently identified, we identified 364 downregulated and 432 upregulated proteins following treatment (FDR > 0.01, FC > 2) (Fig. 6A; Supplementary Table 4). We also noted a significant overlap between transcripts and proteins downregulated by FB23-2 (*P* < 0.001) (Supplementary Fig. 3B). Gene Ontology enrichment analysis again revealed a significant enrichment in cell cycle terms for pathways downregulated by FB23-2 (Fig. 6B). Out of the 61 cell-cycle regulators which were dysregulated at the protein level, 45 were also downregulated on the transcript level (74%) (Fig. 6C, D).

Lastly, we performed western blotting on FB23-2-treated cells to validate our findings and further investigate apoptotic markers and cell cycle phosphorylation events. We again found a substantial decrease in PLK1, CDK2, CCNE2, CCNB1 expression and an increase in CDKN1A (p21) levels upon FB23-2 treatment (Fig. 6E; Supplementary Fig. 3C; Supplemental Material). Additionally, we found a significant reduction in Rb phosphorylation and an increase in cleaved caspase 3 (Fig. 6E; Supplementary Fig. 3C). Overall, these results indicate that FB23-2 treatment leads to significant changes to cell cycle progression in DMG cells, along with the induction of apoptosis.

## Discussion

DMG derives from an OPC lineage and exhibits an aberrant epigenomic landscape driven by H3K27M mutations or EZHIP overexpression [4, 6, 10, 13, 38]. Despite evidence of epigenomic and epi-transcriptomic crosstalk [33–36] and the known roles of m6A in OPC identity [56] the epitranscriptome of DMG has not yet been systematically characterized. Here, we present the first m6A maps of DMG, revealing elevated m6A levels compared to a non-neoplastic, patient-derived brain cell culture, with enrichment on transcripts associated with motility and migration. Conversely, a subset of hypomethylated sites in DMG were enriched for chromosome segregation and cell cycle genes, potentially reflecting the rapid proliferation of DMG cells and increased FTO activity m6A is also known to regulate OPC differentiation and NSC self-renewal, partly by modulating transcripts linked to histone modifications [55, 56]. Consistently, we find that DMG cells retain OPC-like identity, with elevated m6A levels across most transcripts, including histone modification genes and OPC lineage factors. Many of these transcripts also exhibited altered m6A levels following FTO inhibition with FB23-2.

Functionally, FTO inhibition induced a pronounced S-phase accumulation, decreased cell survival, increased apoptosis, and extensive transcriptomic and proteomic changes in DMG cultures. These results parallel the observations made in AML treated with either FB23-2 [51], Brequinar [59], or Bisantrene [59]. Based on our results, we hypothesize that these effects reflect inadequate FTO-dependent regulation of key cell cycle components, particularly those involved in kinetochore function and chromosome segregation, during rapid cell divisions. This aligns with recent work demonstrating that m6A regulates centrosome integrity in cancer cells [65], as well as cell cycle exit and timing in NSCs [66]. Moreover, YTHDF2 is known to regulate the cell cycle by promoting degradation of transcripts such as *CDK2* [67, 68] and is upregulated in DMG. Collectively, these findings illuminate potential targets and mechanisms underlying FTO sensitivity in DMG and help explain similar phenotypes observed in AML studies that lacked m6A profiling.

From a therapeutic perspective, FB23-2 demonstrates minimal toxicity in mice at doses up to 20 mg/kg [51], and reduced efficiency in patient-derived non-neoplastic cells, supporting FTO inhibition as a potentially tolerable strategy. However, FB23-2 and other potent FTO inhibitors currently in development are not predicted to cross the blood-brain barrier [51, 53, 69]. Our findings highlight the urgent need for brain-penetrant FTO inhibitors or alternative delivery methods, such as nanoparticle-based formulations or convection-enhanced delivery. However, careful consideration is warranted, as our data also link m6A modifications to transcripts involved in motility and invasion.

Ultimately, this study advances our understanding of DMG epitranscriptomics, an unexplored facet of its biology, and establishes a foundation for identifying novel therapeutic targets. These include FTO, YTHDF2, as well as specific m6A-modified transcripts involved in cell cycle or invasion, all of which can inform strategies for more effective treatments for this devastating cancer.

## Materials and methods

### Cell culture

SU-DIPGXIII and K27M-KO isogenic cells were maintained adherently on flasks coated with poly-L-ornithine (0.01%) (Sigma) and laminin (10 µg/mL) (Sigma), in Neurocult NS-A proliferation media (StemCell Technologies) supplemented with heparin (0.0002%), EGF (20 ng/mL) and bFGF (10 ng/mL) (StemCell Technologies). SU-DIPGXVII and HSJD-DIPG007 cells were maintained as neurospheres in Tumor Stem Cell (TSM) media consisting of a 1:1 mixture of DMEM/F12 and Neurobasal medium (Invitrogen) with Glutamax, Pyruvate, non-essential amino acids, HEPES buffer, antibiotic/antimycotic, B-27 (Invitrogen), Heparin (0.0002%), EGF (20 ng/mL), bFGF (20 ng/mL), PDGF-AA (10 ng/mL) and PDGF-BB (10 ng/mL) (StemCell Technologies). P000302 cells were maintained adherently in TSM media supplemented with 10% FBS. All lines were cultured at 37 °C and 5% carbon dioxide. Cells tested negative for mycoplasma contamination and were confirmed to match their original tumors by STR profiling.

### Western blotting

Cells were seeded in 6-well plates (2500 cells/well) and 48 h later treated with either DMSO or 8 µM of FB23-2 (Selleckchem). Following 24 h, cells were collected and lysed with RIPA buffer (CST) containing protease and phosphatase inhibitors (Roche) for 15 min on ice with occasional vortexing, followed by centrifugation at 13,000 rpm for 15 min at 4 °C to remove debris. Protein samples (25 µg) were prepared with 1X Laemmli SDS loading dye and then boiled for 5 min at 95 °C. BioRad 4-20% proTEAN pre-cast gels were used for gel electrophoresis, and proteins were transferred for 1 h at 100 V onto 0.45 µm PVDF membranes. Membranes were blocked for 1 h in 5% BSA diluted in Tris-Buffered Saline with 0.01% Tween-20 (TBS-T). Western blotting was carried out overnight at 4 °C with the following primary antibodies (all from CST) diluted at 1:1000 in blocking buffer (CDK2 #2546, Cyclin E2 #4132, Cyclin B1 #4138, PLK1 #4513, p-Rb S807/811 #8526, total Rb #9039, p21 #2947, cleaved caspase 3 #9664, GAPDH #2118, β-actin #8457). Following washing, membranes were incubated in horseradish peroxidase- conjugated secondary antibody (CST) diluted in blocking buffer (1:2000) for 1 h at room temperature. Membranes were incubated with ECL substrate (Pierce) and bands were imaged using a Bio-Rad ChemiDoc imaging system. Densitometry was performed using Image Lab software, with local background subtraction, and normalization to the indicated housekeeping gene. Experiments were performed 3 times. An unpaired *t* test was used to determine significance.

### Cell viability assays

Cells were cultured in 96-well plates at a density of 2500–3500 cells per well and were treated with FB23-3 or STM2457 (Selleckchem) at the indicated concentrations. Adherent cells were treated 24 h after plating, while suspension cells were treated 72 h after seeding. After 72 h of treatment, cell proliferation was evaluated using the resazurin-based Alamar blue assay (Sigma-Aldrich). The resulting data was expressed as a percentage relative to the untreated cells. Experiments were performed 3 times. IC50 values were calculated using GraphPad Prism non-linear regression.

### Clonogenic assays

For adherent colony formation assays (SU-DIPGXIII), 1200 cells were seeded on poly-L-ornithine and laminin-coated 6-well plates. After 72 h, cells were treated with FB23-2 at the indicated doses. For suspension neurosphere colonies, SU-DIPGXVII cells were plated in 24-well plates on a base layer of 0.5% agar in cell culture media. The base layer was then overlaid with a layer consisting of cells (1000 cells/well), 0.33% agar, cell culture media, and FB23-2 at the indicated concentrations. Colonies were allowed to form over a 11 day period, after which they were stained with 3-(4,5-dimethylthiazol-2-yl)-2,5-diphenyltetrazolium bromide (MTT) solution for 1 h at 37 °C. Images of the colonies were captured using Image Lab Software. Colony size and number quantification were performed using Image J software, and the data were presented as a percentage relative to untreated colonies. Experiments were performed 3 times, and an ordinary one-way ANOVA test with Dunnett’s multiple comparisons was used to test for statistical significance between untreated and each dose tested.

### Cell cycle assays

Cells were seeded in 6-well plates (250,000 cells/well) and, following 24 h were treated with the indicated doses of FB23-2. Three wells were pooled for each treatment group. Following 24 h of treatment, cells were dissociated into single cells with accutase, fixed in 1 mL of ice-cold ethanol added drop-wise with continuous vortexing. Cells were fixed for 2 h on ice, washed in PBS, and stained with propidium iodide (10 µg/mL, Sigma #P4864) and RNAse (4 µg/mL, Roche) in PBS. Flow cytometry was performed using a Canto II cytometer and analyzed using FlowJo software. Experiments were performed 3 times, and a 2way ANOVA with Dunnett’s multiple comparisons test was used to test for significance within each cell cycle stage, comparing the different treatment groups to the untreated control.

### Apoptosis assays

SU-DIPGXIII and SU-DIPGXVII were seeded and treated at the same doses as used in the cell cycle assay. After 24 h of treatment, all cells, including those in suspension, were harvested and dissociated into single cells. For each treatment group, cells from three wells were pooled. The collected cells were washed twice with cold PBS prior to performing the Annexin V apoptosis detection assay (BD Biosciences, Cat. No. 556547), following the manufacturer’s instructions. Flow cytometric analysis was conducted using a MACSQuant VYB flow cytometer, and data were analyzed with FlowJo software. Three controls were included to set up compensation and gating: unstained cells, cells stained with FITC Annexin V only, and cells stained with 7AAD only. The experiment was performed three times, and statistical significance between treatment groups and the untreated control was determined using an ordinary one-way ANOVA test for each cell population stage.

### siRNA knockdown

SU-DIPGXIII cells were seeded at a density of 70,000 cells per well 24-well plates coated with poly-L-ornithine (0.01%) (Sigma) and laminin (10 µg/mL) (Sigma) in antibiotic-free Neurocult NS-A proliferation media (StemCell Technologies) supplemented with heparin (0.0002%), EGF (20 ng/mL) and bFGF (10 ng/mL) (StemCell Technologies). The following day, media was changed to DMEM/F12 (Gibco) supplemented with 10% FBS. siRNA constructs (Dharmacon) were transfected into the cells using DharmaFECT-1 transfection reagent (0.6 µL/well) according to the manufacturer’s instructions at a concentration of 50 nM siRNA. siRNA used were ON-TARGETplus Non-targeting Control Pool (D-001810) and ON-TARGETplus FTO (79068). Culture media was changed 4 h following transfection. Cells were harvested following 72 h for qPCR, or monitored for 6 days by Incuyte S3 Live Cell Analysis System.

### Timelapse microscopy proliferation assay

SU-DIPGXIII cells were transfected with siRNA in duplicate wells, and following transfection for 4 h, media was replaced with media containing DMSO or FTO (4 µM). Cells were grown for 6 days, and phase-contrast confluence was monitored by time-lapse microscopy every 6 h using an Incuyte S3 Live Cell Analysis System. Incucyte experiments were repeated three times.

### qPCR

Total RNA was extracted using the ISOLATE II RNA Mini Kit (Meridian Bioscience), and first-strand cDNA was synthesised using the SensiFAST cDNA Synthesis Kit (Meridian Bioscience). Quantitative PCR (qPCR) was performed with the SensiFAST SYBR No-ROX Kit (Meridian Bioscience) using primer sequences listed below. synthesised by Integrated DNA Technologies using a QuantStudio 3 qPCR machine. Relative mRNA expression of genes of interest were normalized to corresponding housekeeping genes. qPCR experiments were performed in biological triplicate.

Sequences of qPCR primers

**Table:** 

Gene	Forward	Reverse
RPS18	GAGGATGAGGTGGAACGTGT	TCTTCAGTCGCTCCAGGTCT
FTO	ACTGACTGGTGGTGTCAACC	AGGCAAGGATGGCAGTCAAG
ALKBH5	GCCTATTCGGGTGTCGGAAC	CTGAGGCCGTATGCAGTGAG
METTL3	AGCCTTCTGAACCAACAGTCC	CCGACCTCGAGAGCGAAAT

### Direct RNA-seq

Direct RNA-seq libraries were prepared by the Garvan Sequencing Platform and sequenced on PromethION flow cells using the SQK-RNA002 kit for SU-DIPGXIII samples and SQK-RNA004 for the SU-DIPGXVII (treated and untreated) and P000302 samples.

### Direct RNA-seq analysis

For RNA004 samples, direct RNA-seq libraries were base-called and m6A-called using Dorado and the rna004_130bps_hac@v5.1.0_inosine_m6A@v1 model (--min-qscore 10 --mm2-opts “-k 14 -w 10 --secondary=no”) and modkit PILEUP. Differentially modified regions were calculated using modkit DMR PAIR. Scatterplots, boxplots, and violin plots were generated using the modkit DMR output, plotting the average m6A level across replicates at sites with aggregated coverage >20 and >10% m6A/A in at least one experimental group. Gene enrichment analysis was performed by calculating the average Δm6A for filtered sites within each gene, ranking the genes based on these values, and then analyzing them using the clusterProfiler package [70]. For RNA002 samples, libraries were base-called and m6A levels calculated using SingleMod-v1 [71].

### RNA-seq

SU-DIPGXIII, SU-DIPGXVII, HSJD-DIPG007 cultures were treated with ~IC50 doses of FB23-2 (8 µM for SU-DIPGXIII and SU-DIPGXVII, 10 µM for HSJD-DIPG007) or DMSO for 24 h before total RNA was extracted using the ISOLATE II RNA Mini Kit from Bioline. cDNA libraries were prepared at the Ramaciotti Center for Genomics using the Illumina Stranded mRNA prep kit. SU-DIPGXIII and HSJD-DIPG007 were sequenced with 100 bp paired-end reads on the NovaSeq 6000 S1 platform, and SU-DIPGXVII was sequenced with 150 bp paired-end reads on the NovaSeq X plus platform. Three biological replicates were performed for all RNA-seq experiments with a 50 million read minimum for all samples.

### RNA-seq analysis

RNA-seq reads were trimmed using Trimmomatic [72] and aligned to the GRCh38 genome using STAR [73]. Quantification of transcript and gene abundance was then performed using RSEM [74]. Differential expression analysis was performed using Degust (10.5281/zenodo.3258932) with Voom/Lima [75], with lowly expressed transcripts filtered with a cutoff of 1 counts per million (CPM) in at least 3 samples. Volcano plots were generated using ggplot2 and the output from Voom/Limma analyses. Gene enrichment analysis on the individual cell lines was performed using the clusterProfiler package [70], and GSEgo function using the log2FC values from all genes. Gene enrichment analysis of commonly dysregulated genes was performed using gprofiler2 [76]. Hypergeometric tests were used to determine if the overlap between upregulated and downregulated genes for SU-DIPGXIII and SU-DIPGXVII was significant. Heatmaps were generated using Pheatmap.

### Mass spectrometry

Mass spectrometry runs were performed by the Bioanalytical Mass Spectrometry Facility (UNSW) with 90 min LC gradient runs. Peptide and protein abundances were calculated using MaxQuant (Min. peptides 1; Label min. ratio count 2; Max mods in site Supplementary Table 3) [77]. Differentially abundant proteins were determined using Perseus (log2 transformation; missing values imputed from normal distribution) and Welch’s *T* test (S0 1; FDR 0.01) [78]. Gene enrichment analysis was performed using the clusterProfiler package and GSEgo function [70]. Heatmaps were generated using Zscores and log2FC values using the Pheatmap package in R.

## Supplementary information

Supplemental Figures

Uncropped western blots

Supplemental Tables

## Data Availability

Data generated in this study have been deposited to European Nucleotide Archive (ENA) under the accession PRJEB80588 and processed data at the Gene Expression Omnibus (GEO) under accession GSE279022. Proteomic dada has been deposited to Proteomics Identification Database (PRIDE) under accession PXD058793.
