# Supplementary material for: RNA N6-methyladenosine (m6A) regulates cell cycle progression in diffuse midline glioma (DMG) and confers sensitivity to FTO inhibition
Source: Cell Death Dis. 2026 Mar 26;17(1):371. doi: 10.1038/s41419-026-08647-8 (PMC13039948; doi:10.1038/s41419-026-08647-8)
Supplement: Supplementary file 1 — Supplemental Figures [file 41419_2026_8647_MOESM1_ESM.pdf]

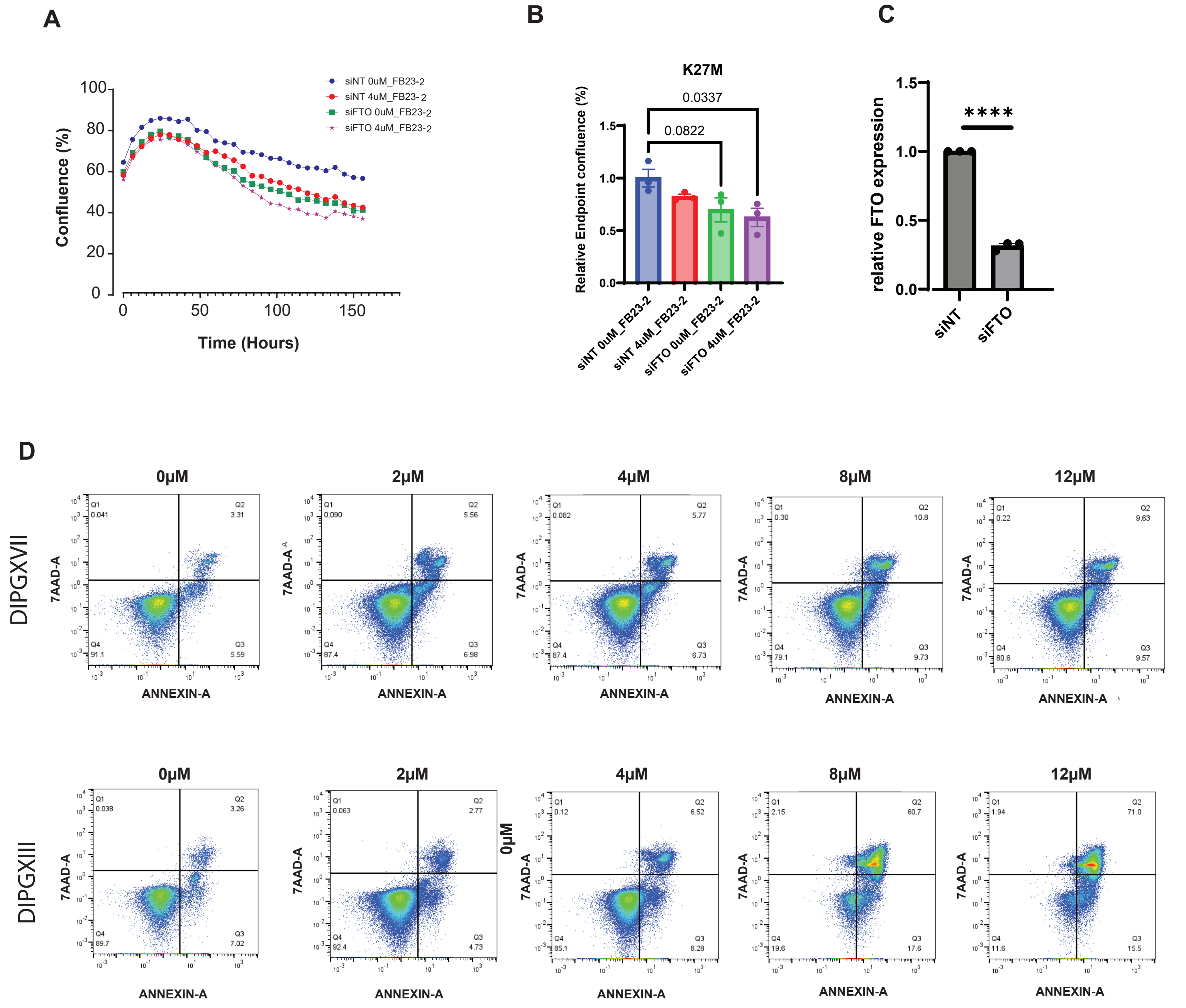


**Supplemental Figure 1. A**) Representative image from the time-lapse proliferation assay of SU-DIPGXIII cells transfected with FTO-targeting siRNA or a non-targeting control (NT), cultured with DMSO or 4 μM FB23-2. Phase-contrast confluence was monitored every 6 h using an Incucyte S3 Live-Cell Analysis System. **B)** Endpoint confluency (~7days) of siRNA proliferation assays, relative to non-targeting (NT) control in DMSO. Data is represented as the mean of 3 biological replicates with standard error shown. **C)** relative FTO expression levels in SU-DIPGXIII cells transfected with FTO-targeting or non-targeting siRNA, after 7 days of culture. Data is represented as the mean of 3 biological replicates with standard error shown. RNA taken from the cells used in proliferation assays. **D)** Flow cytometry analysis of apoptosis after 24 hours of treatment with FB23-2 (0-12 μM) in SU-DIPGXVII (top) and SU-DIPGXIII (bottom).

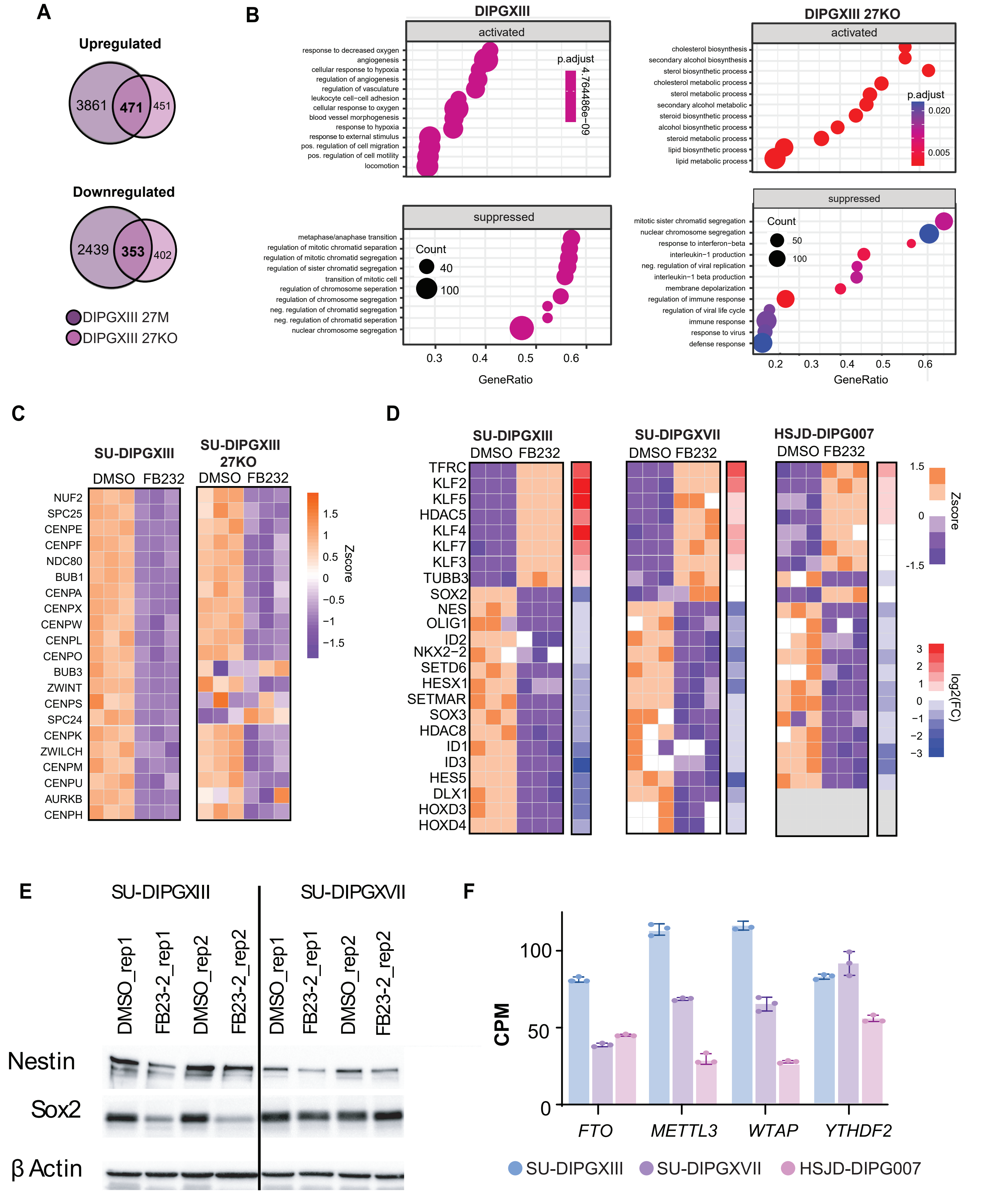


**Supplementary Figure 2. A)** Overlap between genes differentially downregulated and upregulated in SU-DIPGXIII and its isogenic control (K27KO) after FB23-2 treatment. **B)** Gene Set Ontology Enrichment analysis of significantly upregulated and downregulated in SU-DIPGXIII and its isogenic control (K27KO) after FB23-2 treatment. **C)** Heatmap of Zscore values for core kinetechore components expression in SU-DIPGXIII and its isogenic control (K27KO) after FB23-2 treatment. **D) h**eatmap of log2FC and Zscore values for lineage factors which were significantly changes following FB23-2 treatment in atleast one DMG line and with similar trends across lines.**E)** Western blot analysis of Nestin and Sox2 expression following FB23-2 treatment in DIPGXIII and DIPGXVII. **F)** Expression levels (CPM) of FTO, METTL3, WTAP and YTHDF2 in untreated DMG culutres. Data is represented as mean of 3 biological replicates. Error bars represent standard error

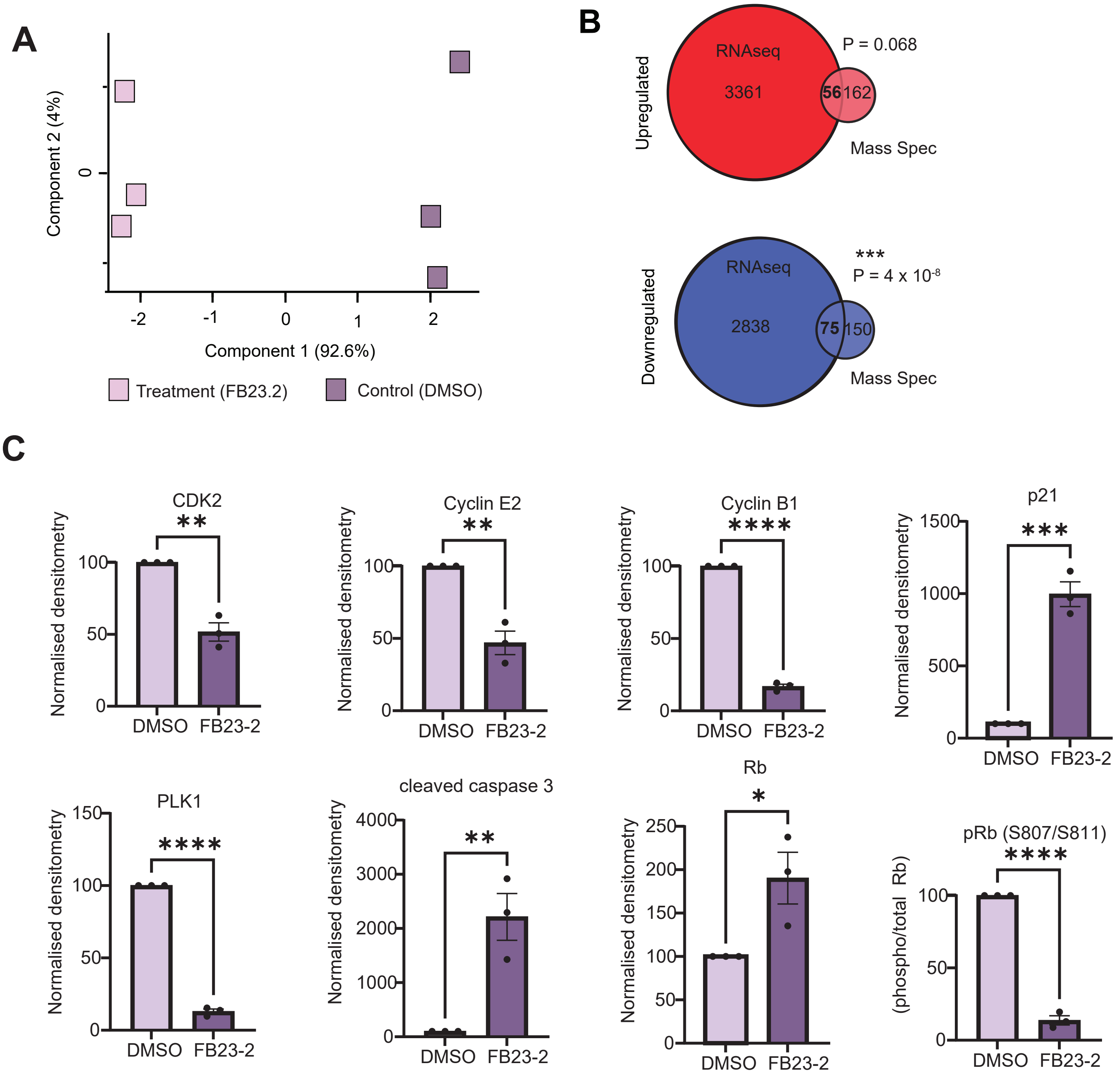


**Supplemental Figure 3. A)** Principal Component Analysis of protein levels in SU-DIPGXIII cells after 24 hour treatment with either DMSO or 8μM FB23-2 treatment. **B)** Overlap between transcripts and proteins differentially upregulated (top) and downregulated (bottom) in SU-DIPGXIII after FB23-2 treatment. **C)** Densitometry analysis of protein levels following 24 hour treatment of 8μM FB23-2 in SU-DIPGXIII. pRB = phosphorylated Rb (retinoblastoma protein); S807/811= serine 807/811. p<0.05, **p<0.01, ***p<0.001, ****p<0.001.
