# Supplementary material for: RNA N6-methyladenosine (m6A) regulates cell cycle progression in diffuse midline glioma (DMG) and confers sensitivity to FTO inhibition
Source: Cell Death Dis. 2026 Mar 26;17(1):371. doi: 10.1038/s41419-026-08647-8 (PMC13039948; doi:10.1038/s41419-026-08647-8)

Fig S2E – Nestin

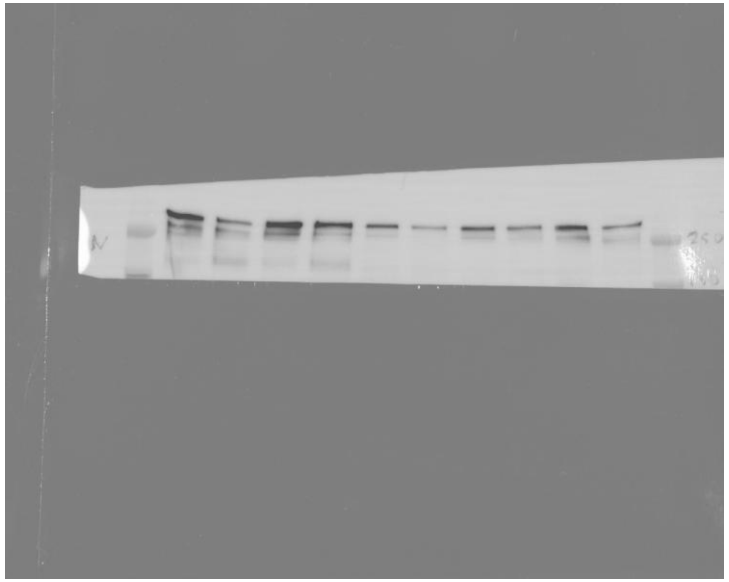

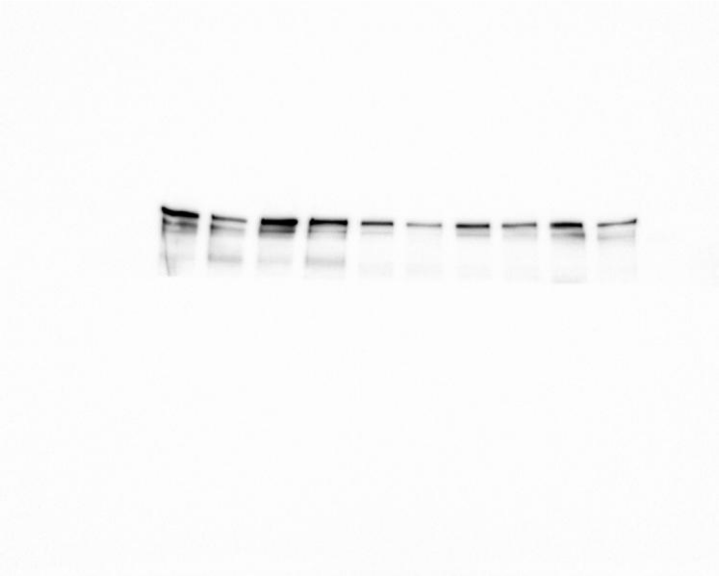

Fig S2E – SOX2

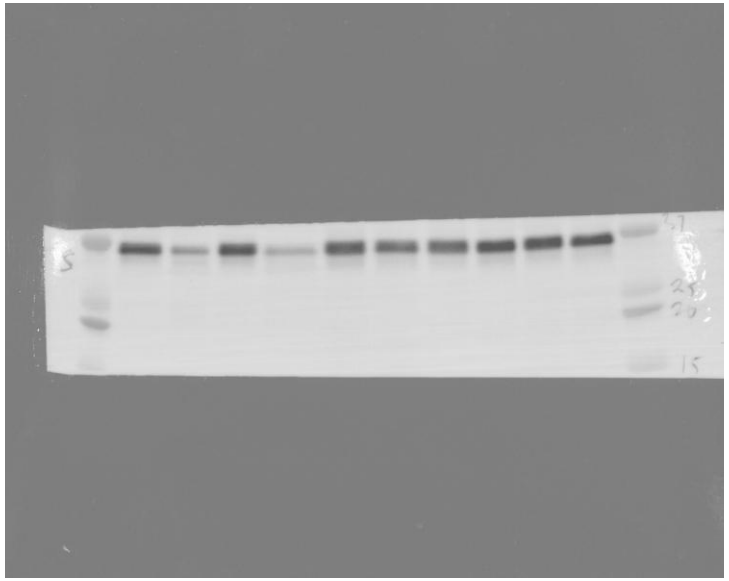

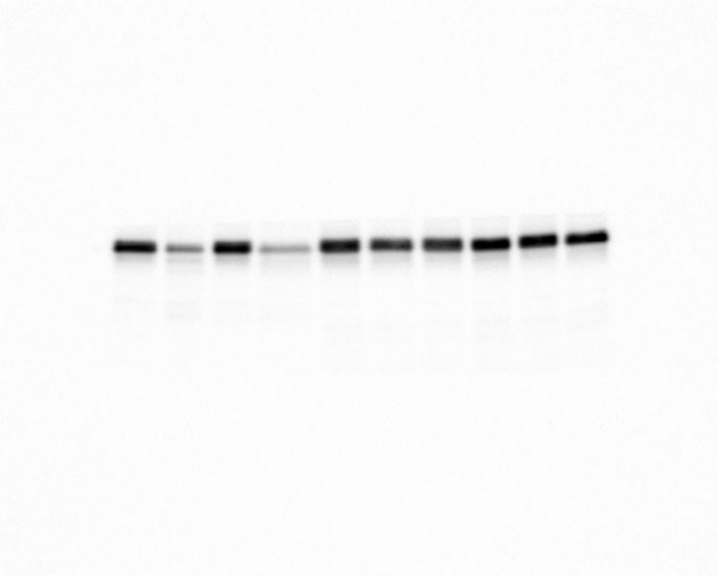

Fig S2E – beta actin

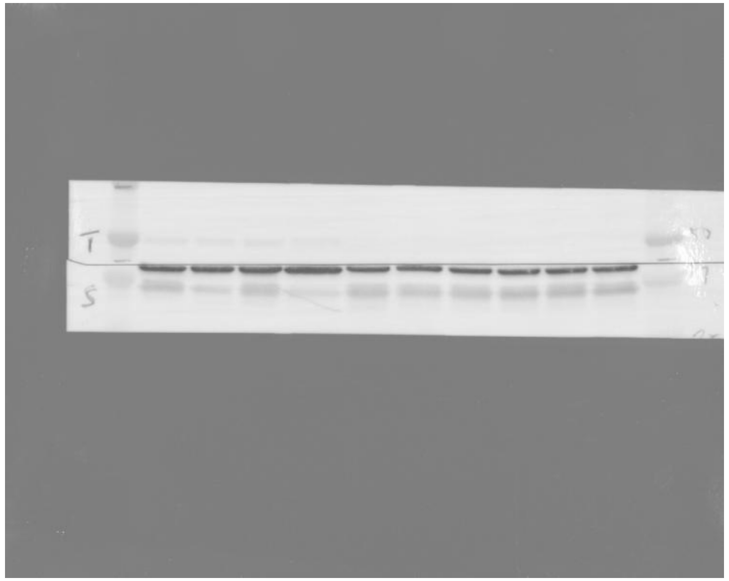

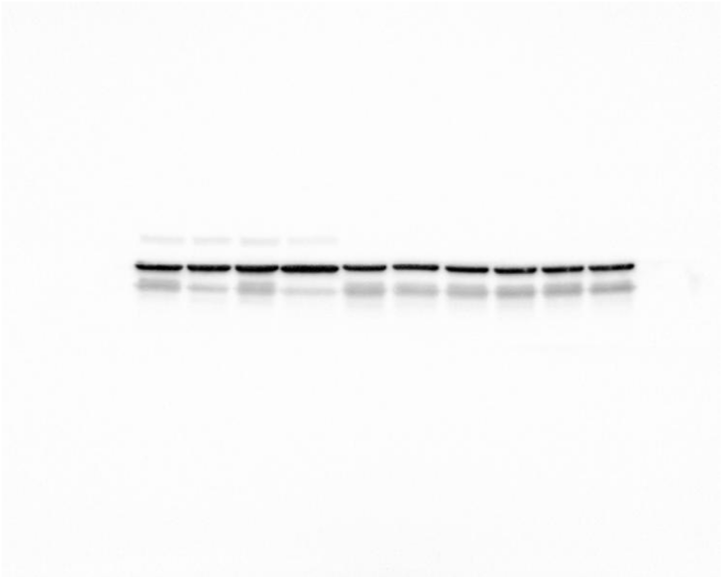

Figure 6E – CDK2

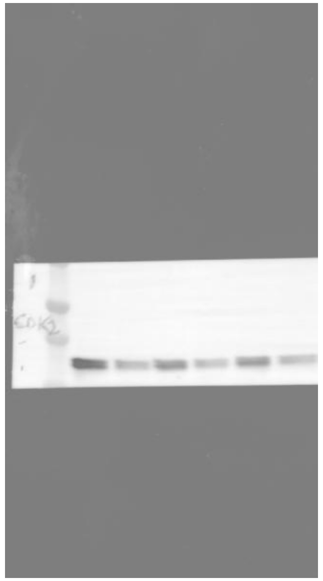

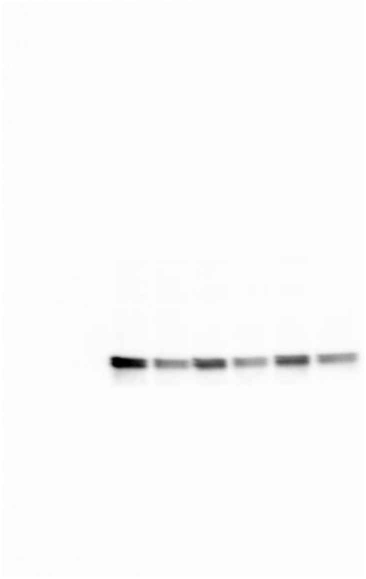

Figure 6E – CCNE2

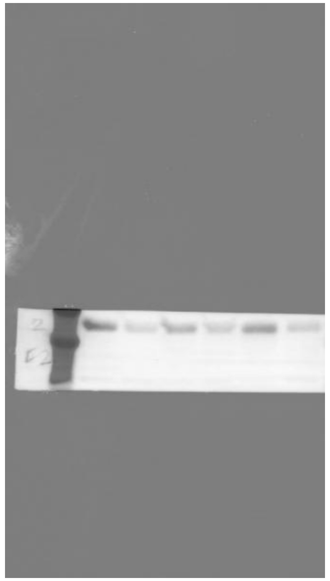

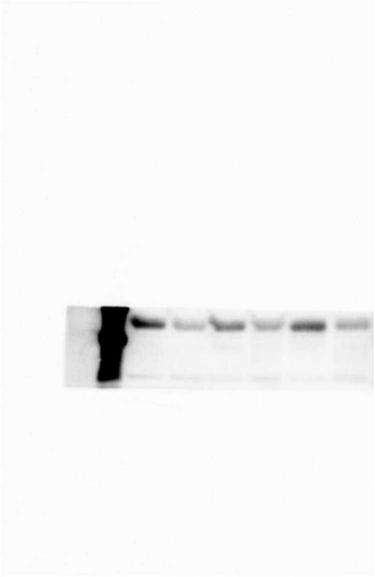

Figure 6E – CCNB1

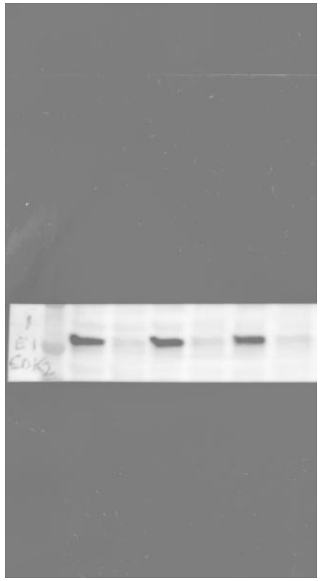

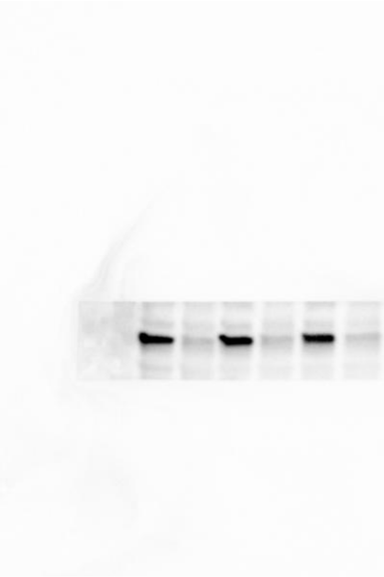

Figure 6E - pRB

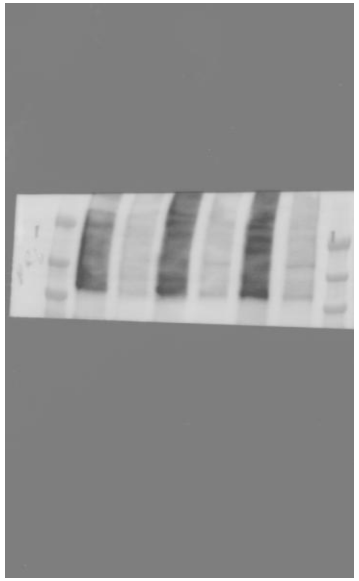

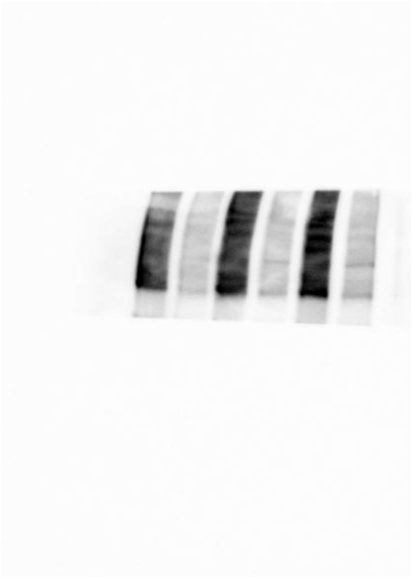

Figure 6E – total Rb

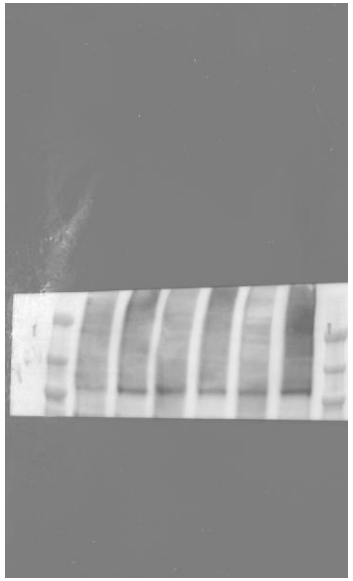

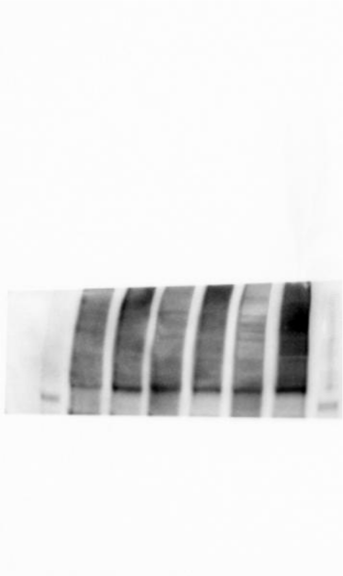

Figure 6E – p21

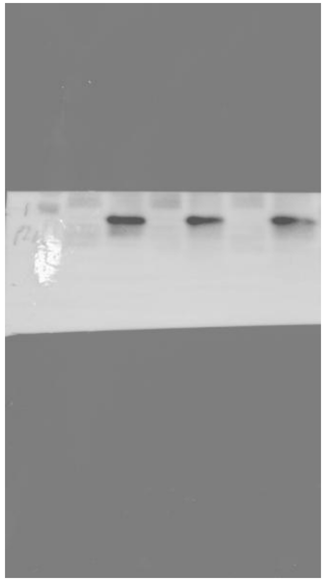

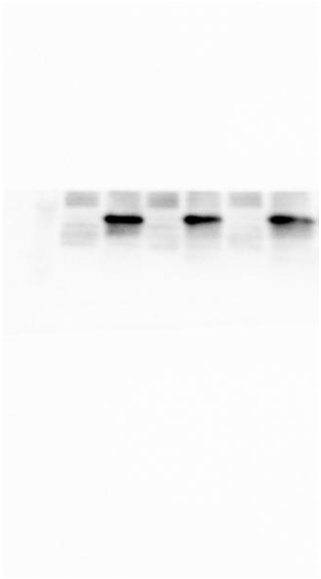

Figure 6E – PLK1

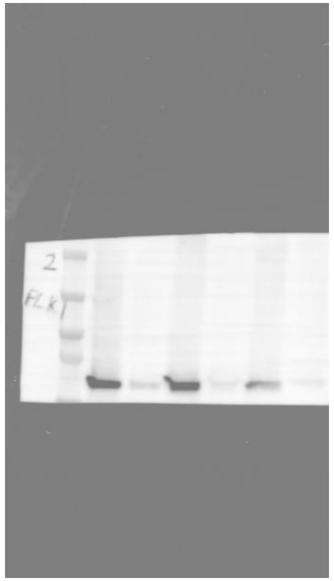

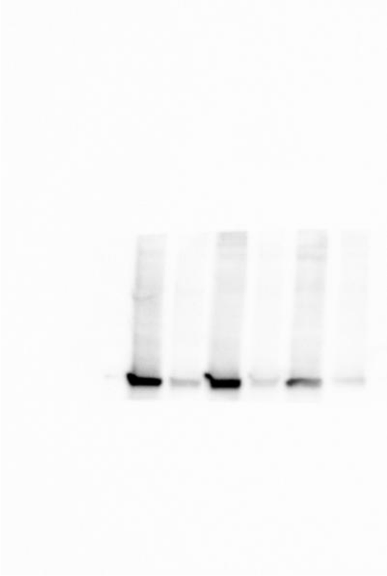

Figure 6E – cleaved caspase 3

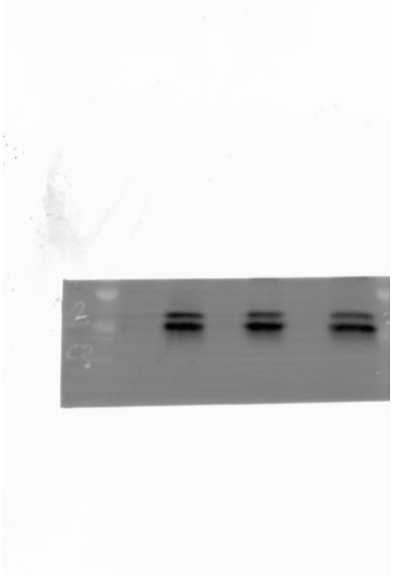

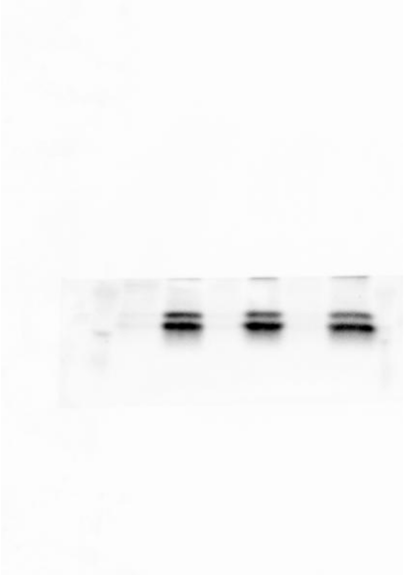

Figure 6E – GAPDH

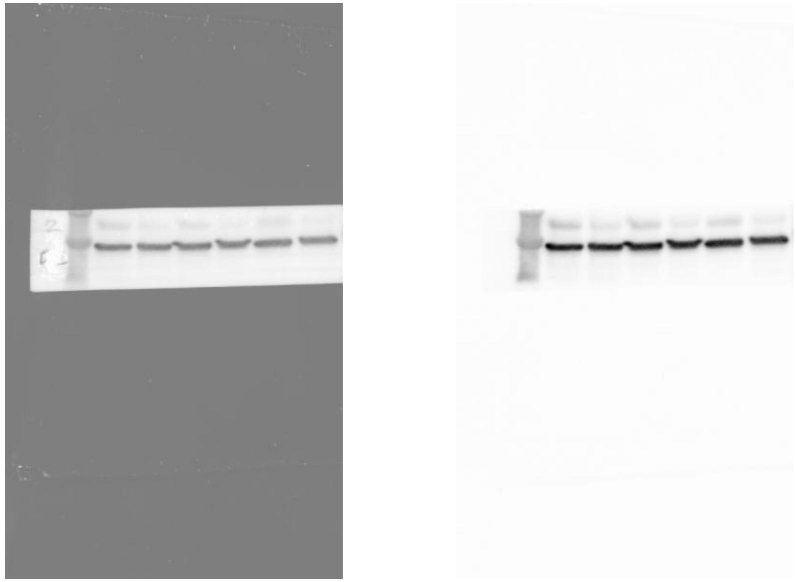

Figure 6E – beta actin

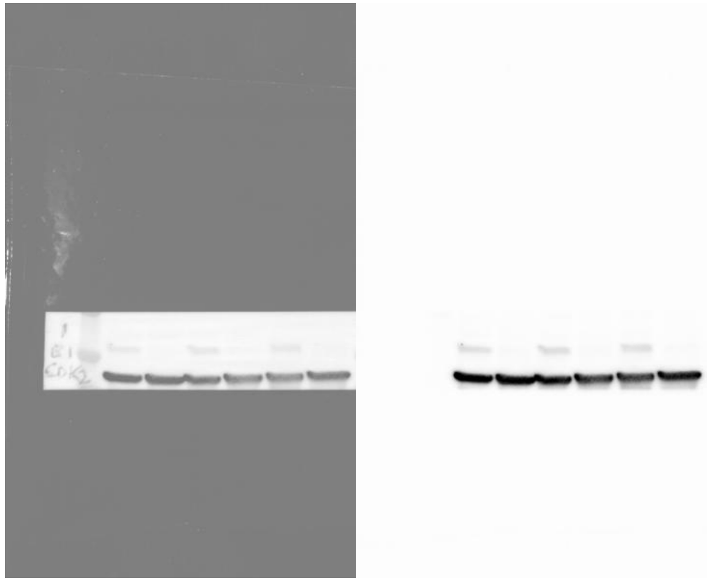

Supplement: Supplementary file 2 — Uncropped western blots [file 41419_2026_8647_MOESM2_ESM.pdf]
